# Storax Protected Oxygen-Glucose Deprivation/Reoxygenation Induced Primary Astrocyte Injury by Inhibiting NF-κB Activation *in vitro*

**DOI:** 10.3389/fphar.2018.01527

**Published:** 2019-01-11

**Authors:** Meng Zhang, Yan Ma, Lijuan Chai, Haoping Mao, Junhua Zhang, Xiang Fan

**Affiliations:** ^1^ Tianjin State Key Laboratory of Modern Chinese Medicine, Tianjin University of Traditional Chinese Medicine, Tianjin, China; ^2^ School of Chinese Materia Medica, Tianjin University of Traditional Chinese Medicine, Tianjin, China; ^3^ Second Affiliated Hospital of Tianjin University of Traditional Chinese Medicine, Tianjin, China; ^4^ Institute of Traditional Chinese Medicine, Tianjin University of Traditional Chinese Medicine, Tianjin, China

**Keywords:** storax, astrocyte, ischemia/reperfusion, NF-κB, ischemic stroke

## Abstract

Stroke is the second leading cause of death and the leading cause of long-term disability in the world. There is an urgent unmet need to develop a range of neuroprotective strategies to restrain the damage that occurs in the hours and days following a stroke. Storax, a natural resin extracted from injuring *Liquidambar orientalis* Mill, has been used to treat acute stroke in traditional Chinese medicine for many centuries. Storax has demonstrated the neuroprotective effects in cerebrovascular diseases. However, the neuroprotective mechanisms activated by storax in ischemia/reperfusion-injured astrocytes have not been elucidated. In this study, we established an oxygen-glucose deprivation/reoxygenation (OGD/R)-induced astrocytes injury model to investigate the effects of storax on OGD/R-induced astrocytes injury and potential mechanisms. Experimental results showed that storax alleviated expression of inflammatory cytokines and protected primary cortical astrocytes injured by OGD/R. Furthermore, storax could inhibit NF-κB activation in injured astrocytes by OGD/R and inhibition of NF-κB with Bay-11-7082 obscured the neuroprotective effects of storax. In conclusion, storax alleviated expression of inflammatory cytokines and protected primary cortical astrocytes injured by OGD/R, which was partially mediated by NF-κB signaling pathway activation.

## Introduction

Stroke is the second leading cause of death and the leading cause of long-term disability in the world. The pathogenesis of stroke-medicated ischemia/reperfusion injury is a complex interplay of aberrant and cascade events: excitotoxicity, peri-infarct depolarization, inflammation, oxidative stress, and apoptosis are involved as the main mechanisms of ischemia/reperfusion-induced damage (Lo et al., 2003; Moskowitz et al., 2010). Over the past decade, although a huge amount of money has been spent in research and development of stroke therapeutics and our knowledge is greatly enriched in terms of understanding the mechanisms of injury and recovery after stroke, there is a remaining translational barrier between basic research benches and practical clinical use. Intravenous recombinant tissue plasminogen activator (tPA) thrombolytic therapy is still the only FDA-approved emergency treatment for acute ischemic stroke (Hacke et al., 2008). However, low reperfusion rate, high risk of intracerebral hemorrhage, and short treatment time window comprise the major limitations to the clinical application of tPA (Kaur et al., 2004; Yepes et al., 2009). Although different kinds of known drugs (such as antiplatelet drugs, anticoagulants, free radical scavengers and neuroprotective agents) are marketed for stroke therapy, the side effects and unknown mechanism of interaction of those drugs are still the huge challenge in clinical (Sacco et al., 2007; Ginsberg, 2008). It is no doubt that developing neuroprotective medicine is clinically and extremely significant, especially to the treatment at acute phase after stroke.

Meanwhile, traditional Chinese medicine is rich in clinical practice and has been proven to be effective for many centuries, which has the potential to facilitate the developments of new treatments for stroke. Currently, there are more than 100 traditional Chinese patent drugs approved by Chinese National Drug Administration used clinically in China for stroke treatment and prevention (Wu et al., 2007), such as the Storax Pill, a Chinese traditional medicinal prescription, which contains 15 various kinds of traditional Chinese medicines, and storax is thought to play the major role in any physiological effects in this prescription. The balsam (storax) produced as a consequence of injuring *Liquidambar orientalis* Mill has been used to treat acute stroke in traditional Chinese medicine for many centuries. Using gas chromatography and mass spectrometry analysis has found that the major components of storax were free cinnamic acid, styracin (cinnamyl cinnamate), phenylpropyl cinnamate, a resin (storesin) consisting of triterpenic acids (oleanolic and 3-epioleanolic acids) and their cinnamic acid esters, and a volatile oil (Duru et al., 2002; Fernandez et al., 2004; Kim et al., 2008; Lee et al., 2009).

Previous studies demonstrated that storax could attenuate the brain damage and regulate the coagulation function in acute focal cerebral ischemia in rats. Storax significantly attenuated the infarct volumes, hemispheric swelling rates, and neurological deficits; decreased the fibrinogen content; prolonged the prothrombin time; and activated the partial thromboplastin time and the content of nitric oxide in serum in focal ischemic stroke rats (Ni et al., 2011a; Zhou et al., 2015). Storax exerts protective effects on astrocytes and the blood brain barrier in ischemia-reperfusion injury after stroke (Ni et al., 2011b). Also, storax has demonstrated protective effects on the brain microvascular endothelial cell damage induced by oxygen-glucose deprivation/reoxygenation (OGD/R), and the mechanism may be related to decrease the expression of inflammatory cytokines (Zhao et al., 2016).

Storax has demonstrated considerable neuroprotective effects in cerebrovascular diseases. However, the neuroprotective mechanisms of storax on ischemia/reperfusion-injured astrocytes have not been elucidated. In this study, we will establish the OGD/R-induced astrocyte injury model to investigate the protective effects of storax on OGD/R-induced astrocyte injury and its potential mechanisms.

## Materials and Methods

### Materials

Refined storax oil was purchased from Tianjin ZHONGXIN Pharmaceutical Group Limited by Share Ltd Darentang Pharmaceutical Factory and conformed to the standard of China Pharmacopeia (2010 version). Dulbecco’s modified Eagle’s medium/F12 (DMEM/F12), glucose-free DMEM, fetal bovine serum (FBS), HBSS, and 0.25% Trypsin-EDTA were obtained from Gibco (Grand Island, NY, USA). CytoTox-ONETM Homogeneous Membrane Integrity Assay and Cell Counting Kit-8 (CCK-8) were purchased from Promega (Madison, WI, USA) and Dojindo (Kumamoto, Japan), respectively. DCFH-DA and Trizol were obtained from Invitrogen (Eugene, USA). Penicillin and streptomycin were purchased from Beyotime (Shanghai, China). Anti-NF-κB p65, anti-p-IκBα antibody, anti-p-IKK antibody, and anti-β-actin antibody were obtained from Cell Signaling Technology (Danvers, MA, USA). Anti-LaminB1 antibody, anti-iNOS antibody, anti-IL-1β antibody, anti-ICAM-1 antibody, and anti-VCAM-1 antibody were purchased from Abcam Technology (Cambridge, MA, USA). SYBR® Select Master Mix and High Capacity cDNA Reverse Transcription Kits were obtained from Applied Biosystems (Foster City, USA).

### Isolation and Culture of Primary Cortical Astrocytes

Primary astrocytes were prepared from the cortex of newborn Wistar rats (less than 24 h) according to the previous methods (McCarthy and de Vellis, 1980; Fan et al., 2015). All the procedures were performed in accordance with animal welfare regulations of the current international laws. Briefly, cortices were dissected free of meninges and homogenized, then digested in 0.25% trypsin in Ca^2+^/Mg^2+^ free PBS for 10 min at 37°C. After terminating the digestion with 10% FBS, filter out the remaining tissue fragments. The cells were centrifuged at 1,000 revolutions per minute for 10 min and diluted with DMEM/F12 plus 1% penicillin/streptomycin and 1% L-glutamine, then plated into the 75 cm^2^ flasks at a density of 2.0 × 10^5^/cm^2^ and maintained at 37°C in an incubator atmosphere with 5% CO_2_ and 95% air. The medium was replaced every 3 days. When cells were firmly attached to the bottom with 90% confluent, the microglia and oligodendrocytes were detached from flasks by shaking at 260 rpm 37°C for 20 h. The cells were passaged and verified by immunofluorescent staining with GFAP. Under these conditions, more than 95% of the cultured cells were GFAP-positive.

### Establishment of OGD/R-Induced Injury of Astrocytes

To mimic the impairment of astrocytes in ischemic injury *in vivo*, we established the OGD/R model *in vitro* as described previously (Ma et al., 2016). Simply, astrocytes were washed with pre-warmed HBSS twice, and the culture medium was replaced with a glucose-free DMEM, and placed astrocytes in a hypoxic humidified incubator chamber (Stem Cell, Canada) flushed with a gas mixture of 95% N_2_ and 5% CO_2_. After OGD for 4 h, the medium was exchanged by DMEM/F12 with high glucose plus storax (0.1, 1, and 10 μg/ml, respectively) or Bay-11-7082 in a humidified atmosphere with 95% air and 5% CO_2_ at 37°C for 20 h reoxygenation and glucose restoration. The controls were incubated with high glucose DMEM/F12 in normal atmosphere for 24 h. Astrocytes were harvested at 24 h after OGD/R.

### Cell Viability and Neurotoxicity

Cell viability was measured using the commercial cell counting kit-8 (CCK-8) following the manufacturer’s instructions. Briefly, after treatments with storax, a total of 10 μl/well of CCK-8 reagent was added in 96-well plate. After incubation, the absorbance at 450 nm was measured using microplate reader. Lactate dehydrogenase (LDH) release is an indicator of plasma membrane damage, and a commercial LDH assay kit was used for the measurement of neurotoxicity following the manufacturer’s instructions.

### Measurement of Intracellular Reactive Oxygen Species (ROS) Level

Intracellular ROS level was determined as described previously (Ma et al., 2016). Astrocytes were loaded with 5 μM DCFH-DA at 37°C for 30 min and then washed with D-hank’s three times to remove residual probe. The cellular fluorescence was measured with a fluorescence microplate reader at an ex/em wavelength of 488/525 nm. Results were expressed as a percentage of the control group.

### Quantitative Real-Time Polymerase Chain Reaction Analysis

Total ribonucleic acid from astrocytes was extracted using Trizol and was reverse transcribed by TaqMan reverse transcription reagents according to the manufacturer’s instructions. All primers were synthesized by Sangon Biotech (Shanghai, China); the forward and reverse primers for iNOS, IL-1β, IL-6, TNF-α, and NF-κB are described in Table 1. The amplification was accomplished in an ABI 7500 Real-Time PCR System (Applied Biosystems, Foster City, USA). The target gene expression was examined by the 2-ΔΔCT method. Analysis was carried out in triplicates.

**Table 1:** The sense and antisense primers.

Genes	Primer/Probe	Primer/Probe sequences (5ʹ to 3′)
iNOS	Forward primer Reverse primer	5′-GCTACACTTCCAACGCAACA-3′ 5′-ACAATCCACAACTCGCTCCA-3′
IL-1β	Forward primer Reverse primer	5′-GAAGAGACGAGAGATCGA-3′ 5′-CACACAGATCTCCTCAAGGCA-3′
IL-6	Forward primer Reverse primer	5′-ATCACGAACTAGAGCAGCAG-3′ 5′-TCATACACAGCCACAGTCACCAC-3′
TNF-α	Forward primer Reverse primer	5′-GAGTGAGAGATGTAGAGG-3′ 5′-ATACATTAGGGAGAACAACGA-3′
NF-κB	Forward primer Reverse primer	5′-GATAAAATCCTCGGGGTCCTAC-3′ 5′-GCTGCTATGTGTAGAGGTGTCG-3′

### Measurement of NF-κB Deoxyribonucleic Acid Binding Activity

NF-κB DNA binding activity was determined by electrophoretic gel mobility shift assay (EMSA) as previously described (Lee et al., 2008). Briefly, 5 μg of total nuclear proteins was mixed with double-stranded γ [32P] adenosine triphosphate end-labeled oligonucleotides containing consensus binding sequences for NF-κB (sense strand 5′-AGGGACTTTCCGCTGGGGACTTTCC-3′), then incubated in binding buffer for 30 min at room temperature and run through non-denaturing polyacrylamide gel. After exposed to Kodak X-ray film, the signal was detected and quantified by computer-assisted densitometry.

### Measurement of NF-κB Nuclear Translocation

Immunohistochemistry was performed by following a standard method to detect NF-κB nuclear translocation as previously described (Wei et al., 2000). Astrocytes were fixed with paraformaldehyde and then incubated with rabbit anti-NF-κBp65 (1:200, CST, USA) antibody overnight at 4°C following a second antibody Alexa Fluor® 488 nm (1:1000, Life Technology, USA).

### Western Blot Analysis

After washed with ice cold D-hank’s, astrocytes were lysed in RIPA buffer. Protein concentration was determined using the BCA Bradford protein assay kit, and protein samples were mixed with SDS-PAGE loading buffer. After heated at 100°C for 8 min, proteins were subjected to 10–12% SDS-PAGE gel and transferred electrophoretically to polyvinylidene difluoride membranes (Millipore, USA) and then blocked with Tris-buffered saline including 5% nonfat milk and 0.1% Tween-20 for 2 h at room temperature. Incubation with primary antibody against NF-κBp65 (1:1000, CST, USA), p-IκBα antibody (1:1000, CST, USA), p-IKK (1:1000, CST, USA), iNOS (1:1000, Abcam, USA), IL-1β (1:1000, Abcam, USA), ICAM-1 (1:1000, Abcam, USA), VCAM-1 (1:1000, Abcam, USA), β-actin (1:4,000, CST, USA), and LaminB1 (1:1000, Abcam, USA) was done overnight at 4°C and then with HRP-conjugated secondary antibody (1:2000). Antibody positive bands were detected with an enhanced chemiluminescence agent (Millipore, USA). Quantification of bands was made by scanned densitometric analysis and Quantity one analysis system.

### Statistical Analysis

Data were expressed as means ± S.E.M. Comparisons between multiple groups were performed by a one-way analysis of variance (ANOVA). *p* < 0.05 was considered to be significant. All of the data obtained from at least three repeated experiments, and statistical analyses were performed using IBM SPSS Version 21.0 (SPSS Inc., Chicago, IL, USA).

## Results

### Storax Protected Primary Cortical Astrocytes Injured by OGD/R

**Figure 1:**
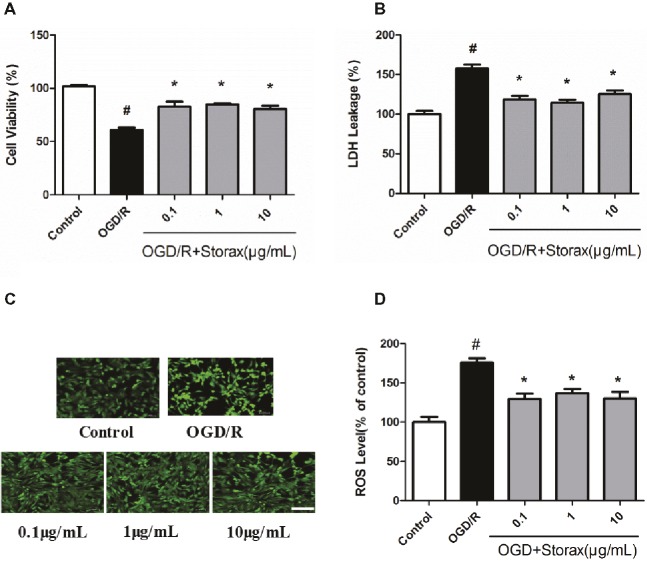
Neuroprotective effects of storax on OGD/R-induced injury in astrocytes. **(A)** Cell viability was measured by CCK-8 assay (*n* = 18). **(B)** LDH release (*n* = 18). **(C)** The intracellular ROS generation was indicated by DCFDA fluorescence. Scale bar, 100 μm. **(D)** Quantitative data analysis of intracellular ROS generation in astrocytes (*n* = 10). Data are expressed as mean + S.E.M. #*p* < 0.05 compared with control; ^∗^ *p* < 0.05 compared with OGD/R.

OGD/R induced 34.83 ± 4.66% reduction in cell viability. Treatment with 0.1, 1, and 10 μg/ml storax improved cell viability compared with OGD/R group (Figure 1A). LDH release was increased following OGD/R, while 0.1, 1, and 10 μg/ml storax suppressed LDH release from astrocytes to culture medium compared with OGD/R group (Figure 1B). OGD/R induced a significant increase in intracellular ROS generation to 63.67 ± 8.0% compared with control group; treatment with 0.1, 1, and 10 μg/ml storax significantly decreased intracellular ROS generation compared with OGD/R group (Figures 1C,D).

### Storax-Alleviated Expression of Inflammatory Cytokines in OGD/R-Injured Astrocytes

**Figure 2:**
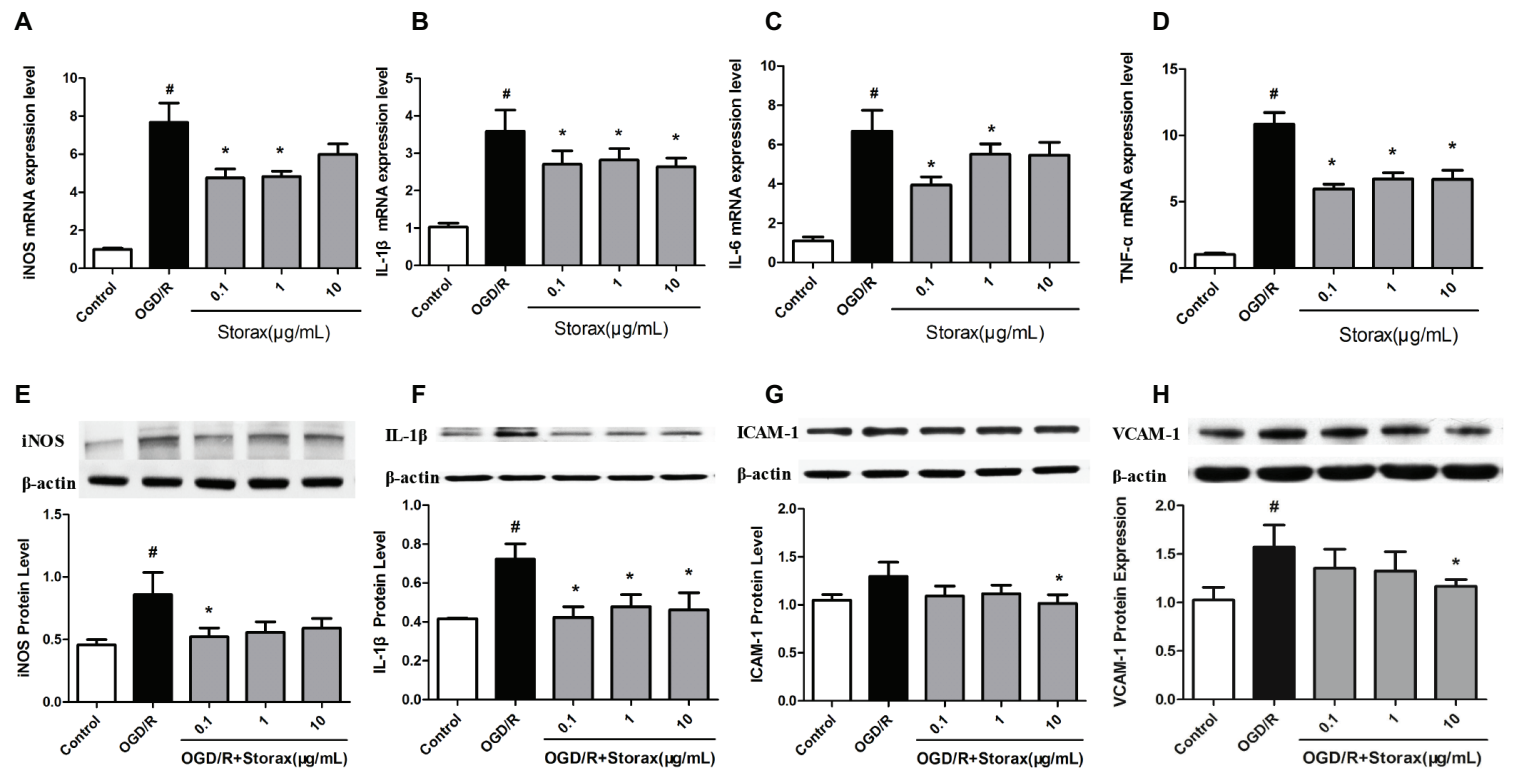
Effects of storax on inflammatory cytokines and adhesion molecules expression in astrocytes following OGD/R-induced injury. **(A)** iNOS mRNA expression detected by real-time PCR (*n* = 10). **(B)** IL-1β mRNA expression detected by real-time PCR (*n* = 10). **(C)** IL-6 mRNA expression detected by real-time PCR (*n* = 10). **(D)** TNF-α mRNA expression detected by real-time PCR (*n* = 10). **(E)** iNOS expression detected by Western blot (*n* = 3). **(F)** IL-1β expression detected by Western blot (*n* = 3). **(G)** ICAM-1 expression detected by Western blot (*n* = 3). **(H)** VCAM-1 expression detected by Western blot (*n* = 3). Data are expressed as mean + S.E.M. #*p* < 0.05 compared with control; ^∗^ *p* < 0.05 compared with OGD/R.

OGD/R injury could trigger inflammation and increase the mRNA expression of iNOS, IL-1β, IL-6, and TNF-α in astrocytes, while 0.1, 1, and 10 μg/ml storax suppressed iNOS, IL-1β, IL-6, and TNF-α mRNA expression compared with OGD/R group (Figures 2A,D). Western blot analysis confirmed that OGD/R enhanced the expression of iNOS and IL-1β in astrocytes, while storax decreased iNOS and IL-1β expression compared with OGD/R group (Figures 2E,F). Additionally, storax could diminish OGD/R induced the increase of ICAM-1 and VCAM-1 expression in astrocytes (Figures 2G,H).

### Storax Inhibited NF-κB Activation in Injured Astrocytes by OGD/R

**Figure 3:**
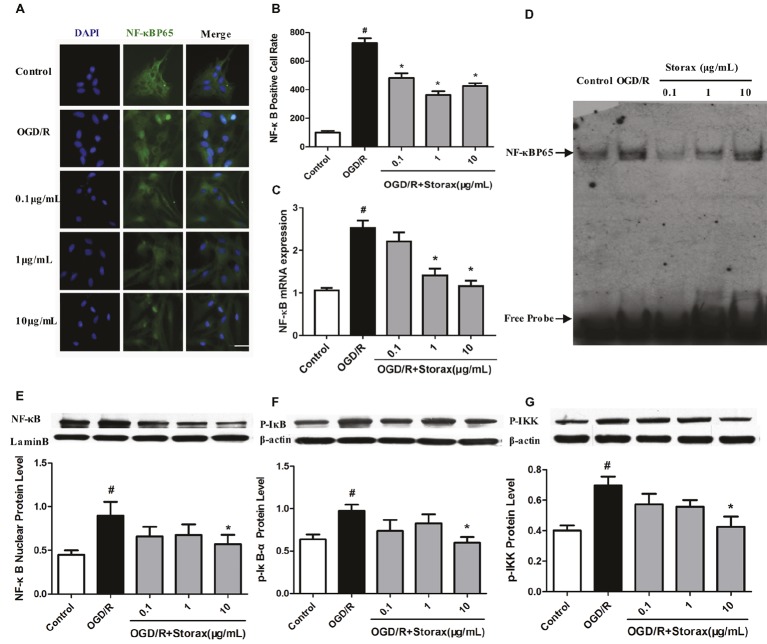
Effects of storax on NF-κB activation in injured astrocytes by OGD/R. **(A)** Representative images of NF-κB p65 nuclear translocation in astrocytes. Scale bar, 50 μm. **(B)** Quantitative data of NF-κB p65 nuclear translocation in astrocytes. **(C)** NF-κB p65 mRNA expression detected RT-PCR (*n* = 10). **(D)** DNA binding activity of NF-κB detected by EMSA. **(E)** Phosphorylation of NF-κB p65 expression detected by Western blot (*n* = 3). **(F)** Phosphorylation of IκB expression detected by Western blot (*n* = 3). **(G)** Phosphorylation of IKK expression detected by Western blot (*n* = 3). Data are expressed as mean + S.E.M. #*p* < 0.05 compared with control; ^∗^ *p* < 0.05 compared with OGD/R.

The effect of storax on NF-κB activation in astrocytes induced by OGD/R was determined using immunofluorescence. OGD/R increased NF-κB p65 nuclear translocation in astrocytes, and treatment with storax decreased nuclear translocation of NF-κB p65 compared with OGD/R group. Compared with the control group, the percentage of NF-κB p65 nuclear translocation was dramatically increased in OGD/R group (693.77 ± 40.43%, *p* < 0.05). This increase was significantly inhibited by storax (Figures 3A,B). OGD/R significantly increased NF-κB p65 mRNA expression in astrocytes, while treatment with storax decreased NF-κB p65 mRNA expression compared with OGD/R group (Figure 3C). Storax treatment decreased the DNA binding activity of NF-κB detected by EMSA after OGD/R (Figure 3D); also, storax treatment inhibited OGD/R-mediated phosphorylation of IκB, NF-κB p65, and IKK detected by western blot (Figures 3E,G).

### Effects of Bay-11-7082 on Cell Viability and Neurotoxicity in OGD/R Injured Astrocytes

**Figure 4:**
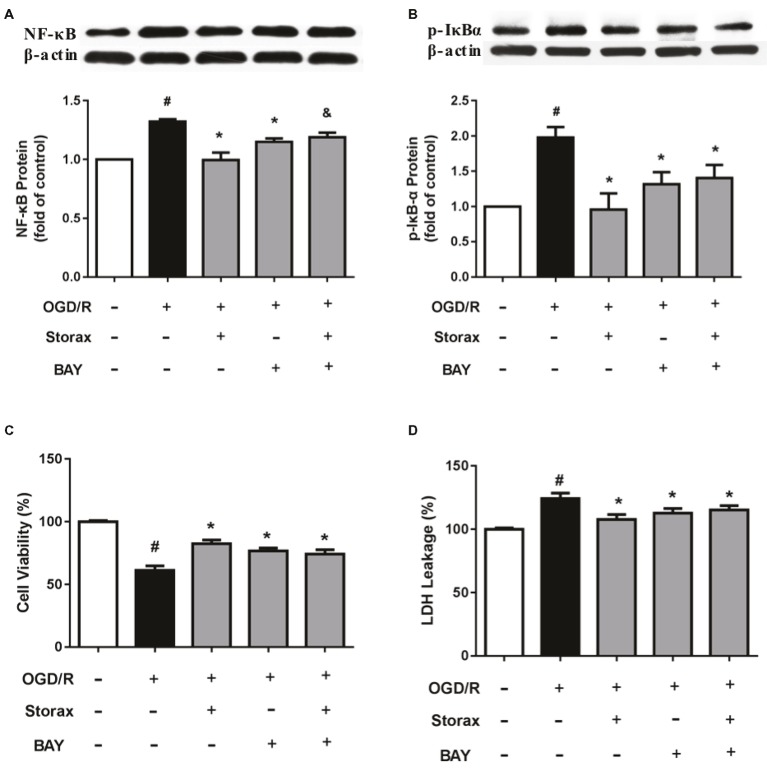
Effects of Bay-11-7082 on cell viability and neurotoxicity in OGD/R-injured astrocytes. **(A)** NF-κB p65 expression detected by Western blot (*n* = 3). **(B)** Phosphorylation of IκB expression detected by Western blot (*n* = 3). **(C)** Cell viability was measured by CCK-8 assay (*n* = 16). **(D)** LDH release (*n* = 16). Data are expressed as mean + S.E.M. #*p* < 0.05 compared with control; ^∗^ *p* < 0.05 compared with OGD/R, and *p* < 0.05 compared with storax treatment.

Bay-11-7082, as NF-κB inhibitor, significantly inhibited activation of NF-κB p65 and p-IκBα protein (Figures 4A,B) and protected astrocytes from OGD/R injury (Figures 4C,D). While pre-treatment with Bay-11-7082 obscured storax decreased activation of NF-κB p65 and p-IκBα protein (Figures 4A,B), and storax induced increase of cell viability and reduction of neurotoxicity compared with storax treatment alone (Figures 4C,D).

## Discussion

Experimental results demonstrate that storax exerts considerable neuroprotective effects by attenuating the expression of a range of inflammatory cytokines on injured primary cortical astrocytes following OGD/R. These protective effects are mediated by an inhibition of NF-κB and can be partially reversed by pre-treatment with Bay-11-7082.

Ischemic stroke triggers a complex cascade of pathophysiologic events in the brain, including excitotoxicity, acidosis, inflammation, oxidative stress, peri-infarct depolarization, and apoptosis (Dirnagl et al., 1999; Lo et al., 2003; Moskowitz et al., 2010), with particularly harmful effects of inflammation and apoptosis, which ultimately leads to cell death and infarction (Dirnagl et al., 1999). The abovementioned processes interact with each other and exert complex patterns of pathology following after cerebral ischemia. Evidence is now accumulating that glial cells, especially astrocytes, play an important role in the pathophysiology of cerebral ischemia. Astrocytes are the most abundant non-neuronal cell type in the central nervous system; neurons cannot survive in the brain without a close interaction with astrocytes. Accordingly, astrocyte function can critically influence neuronal survival following ischemia (Swanson et al., 2004). Therefore, preventing astrocytes from injury and death demonstrates a considerable therapeutic potential to protect the brain following ischemia and reperfusion injury (Zhan et al., 2010). By mimicking ischemia and reperfusion injury, we established an OGD/R-induced astrocyte injury model *in vitro*. In this study, storax exerted protective effects against OGD/R-induced astrocyte injury by improving cell viability and decreasing LDH release (Figures 1A,B). As in mammals, intracellular [Ca_2+_] precedes the surge of ROS levels that appears to be critical for the cerebral ischemia-reperfusion injury, and previous studies suggested that ROS could increase mitochondrial permeability transition pore opening during ischemia-reperfusion (Brookes and Darley-Usmar, 2004). As shown in Figure 1C, OGD/R induced a significant increase in intracellular ROS generation, while storax significantly decreased intracellular ROS generation. These data suggest that storax is able to protect astrocytes from OGD/R injury.

Inflammation plays one of the key roles in the pathology of cerebral ischemic injury. Cerebral ischemia triggers a marked inflammatory response characterized by the release and activation of adhesion molecules, chemokines, and cytokines, which aggravate brain damage (Lo et al., 2003; Moskowitz et al., 2010). Emerging evidence demonstrates that the pro-inflammatory cytokines, such as IL-6, IL-1β, and TNF-α, are the initial triggers of reactive astrocytes in the acute phase of cerebral ischemic injury (Aranguez et al., 1995; Lin et al., 2006). Moreover, reactive astrocytes result in the secretion of different pro-inflammatory cytokines and adhesion molecules, such as ICAM-1, VCAM-1, IL-6, IL-1β, and TNF-α, which may involve in a cyclic process of continuous activation (Cui et al., 2011; Jeong et al., 2012). Therefore, decreasing the inflammatory response induced by activated astrocytes could prevent neuroinflammation after cerebral ischemia. Our study demonstrates that OGD/R injury could trigger inflammation and increase the expression of adhesion molecules and pro-inflammatory cytokines in astrocytes, such as ICAM-1, VCAM-1, IL-6, IL-1β, and TNF-α, while storax suppressed those adhesion molecules and pro-inflammatory cytokine expression (Figure 2). These data suggest that storax could decrease inflammatory cytokines and adhesion molecule release from OGD/R injured astrocytes; furthermore obscured astrocytes activation induced by adhesion molecules and pro-inflammatory cytokines.

NF-κB, as a transcription factor, is important in the inflammatory responses of astrocytes, and NF-κB activation is necessary for the gene transcription of encoding pro-inflammatory cytokines (Yasuda et al., 2011). NF-κB family is comprised of five different members, such as p50, p52, p65, c-Rel, and Rel-B. The phosphorylation of p65 is wildly studied NF-κB activation form, which helps to stabilize NF-κB complex in the nucleus for gene expression in the nervous system (Schmitz and Baeuerle, 1991). The activation of NF-κB initiates with the phosphorylation and the subsequent degradation of inhibitor of κB (IκB), which subsequently causes the translocation of free NF-κB from cytoplasm to nucleus, where it promotes the expression of pro-inflammatory genes (Iadecola and Anrather, 2011). Indeed, inactivation of astrocyte NF-κB signaling decreases cytokine production and protects neurons after ischemic injury (Dvoriantchikova et al., 2009; Barreto et al., 2011). We observed in this study that OGD/R enhanced the degradation of IκBα, NF-κB phosphorylation, and translocation of active NF-κBp65 from the cytoplasm to the nucleus in astrocytes, whereas storax diminished these NF-κB changes (Figure 3). Storax-mediated activity relied on NF-κB activation, confirmed by the use of the NF-κB specific inhibitor BAY11-7082, which prevented the effects of storax on both cell viability and neurotoxicity partially (Figure 4), indicating that NF-κB activation has a key role in storax-mediated protection on OGD/R-injured astrocytes.

There are several caveats and limitations in this work. First, the components of storax are very complex, and the main components include free cinnamic acid, styracin (cinnamyl cinnamate), phenylpropyl cinnamate, a resin (storesin) consisting of triterpenic acids (oleanolic and 3-epioleanolic acids) and their cinnamic acid esters, and a volatile oil. Further experiments to confirm the neuroprotective components of storax would be clinically important. Second, astrocyte function can critically influence neuronal survival during ischemia, whether storax could protect neuron *via* astrocytes and the interaction between neuron and astrocytes, as well as the underlying molecular pathological mechanisms need to be further defined.

Collectively, our study demonstrated that storax alleviated expression of inflammatory cytokines and protected primary cortical astrocytes injured by OGD/R, which was mediated by NF-κB signaling pathway activation. We are aware that it would be very important to identify the neuroprotective key components of storax and the mechanism how it works with NF-κB in future investigations.

## Ethics Statement

This study was carried out in accordance with the recommendations of the Tianjin University of Traditional Chinese Medicine Guide for Care and Use of Laboratory Animals. The protocol was approved by the Committee of Care and Use of Laboratory of Tianjin University of Traditional Chinese Medicine.

## Author Contributions

MZ and XF contributed to the design of the study. MZ, YM, LC, and HM were responsible for the data collection. MZ, YM, and LC analyzed the data. MZ and XF interpreted the data. MZ and XF drafted the manuscript. JZ revised the manuscript content. XF approved the final version of manuscript.

### Conflict of Interest Statement

The authors declare that the research was conducted in the absence of any commercial or financial relationships that could be construed as a potential conflict of interest.
